# Diagnosis and Etiology of Idiopathic Fevers

**Published:** 1900-09

**Authors:** F. M. Brantly

**Affiliations:** Senoia, Ga.


### DIAGNOSIS AND ETIOLOGY OF IDIOPATHIC FEVERS.

BY F. M. BRANTLY, M.D.,
SENOIA, GA.

Causation is the great problem everywhere sought after, and evokes the aid of every department of science. Theology discourses on theoretical imagination, seeking to build facts on immateriality.

A fulcrum is essential to all physical power; in like manner, matter is the foundation upon which science must build, beginning at the concrete and ending in the infinitesimal. Every department of physics has its votaries, among which is found in prominence the disciples of Æsculapius, whose innings are constantly revolutionizing all medical departments and developing the hidden mysteries of nature, bringing to light truth and setting aside cherished and long-lived traditions of the fathers, astounding themselves and the world to see what a day of small things can bring forth. The phantom cure-alls of to-day, who seem to have the world in swing, are gone to-morrow, and others more pretentious come in their places to deceive the credulous; these blatant monstrosities only wait in turn to be knocked out by others, until science shall have demonstrated its triumphs and forever put to rest the calling of the mountebank.

Etiology is a big word and means much; it goes from branch to root; its workings are seen and felt all through the physical stamina, is much talked about but less known. Let us study it in fevers for a few moments.

Fever derives its name from pyrexia—heat, and is said to originate from nervous disturbances, by some. However, nosologists differ widely in their views on the causes that give rise to the excess of heat. In the absence of any settled opinion as to this phenomenon, we would conclude that the combustion going on in the lungs, together with the excessive heart action to eliminate the morbific cause, as does excessive exercise elsewhere, producing friction, results in generating caloric.

Hippocrates, Broussais, Paracelsus, Cullen, Brown, and others were divided in their pathological and etiological views, some contending that all diseases originated in the solids; others that the fluids were the *fons et origo* of all diseases. The two contending parties and their successors fought it out on the two theories for ages, while humanity suffered on account of practice conforming to theory.

Plausible theory that does not conform to fact is a constant menace to humanity. Theory is the life of empiricism and is the hobby of mountebanks. In the absence of knowledge theory always asserts itself, and tenaciously holds its ground until set aside by solid fact, and then it lingers long and dies hard. It is no wonder that medical men disagree because of so limited a knowledge of vital phenomena, and as an excuse for the unknown men will cobble up a theory, sometimes plausible and sometimes a miserable makeshift. Arbitrary nomenclature, doubtless, has as much or more to do in confusing the minds of medical men as any other thing. When you consult the current authors you are constantly confused and confronted with the various names given to the same disease.

Idiosyncrasy and fortuitous circumstances have much to do in these diversities; while the causation is the same, the effects are dissimilar, as the cause may be more or less virulent, and so of the effect; and then temperament has much to do in showing different aspects produced. These accountable or unaccountable phenomena give rise to the unwarrantable and ambiguous names given to diseases arising from the same fundamental cause. We know that vegetable decomposition generating microbes, or miasm imbibed into the general system by the various inlets, produces malarial fevers; and that those fevers, whose names are legion and all amenable to treatment, and *sui generis* and curable by their antidote, quinine, which destroys the plasmodium of malaria, include the quotidian, tertian, quartan, estivo, congestive, algid, etc., all brought about by the same cause and amenable to the same remedies which are known to destroy the parasitic microbes in the blood, whose cycles are said to characterize the stages of pyrexia in all fevers produced by their infesting the corpuscles and plasmodium of the blood. This class of fevers may be all grouped together and offer work for the diagnostician who will not materially err when bearing constantly in mind these suggestions. The habitat of this miasm is almost always found in localities where moisture, warmth and vegetable decay exist. Some contend that the mosquito bite produces the toxic effects of malaria, and that the various kinds produce the various grades of these fevers. But that idea is precluded by the fact that in many places where these parasites abound there are no malarial fevers, and *vice versa.*

The red corpuscles of the blood seem to be the favored pabulum for malarial microbes, and in their evolution produce the sthenic and asthenic changes, and the anemic condition always found early in these fevers and never in typhoid until ten or fifteen days have elapsed. Either from lack of close observation or from sinister motives some medical men are always ready to pronounce synocha or syochus fevers typhoid, or if they apprehend a reversal from any source they compromise on the term typho-malarial, a misnomer, but an easy term to get out on, and, to make their diagnosis more plausible, roundly assert that malarial and typhoid fevers can and do amalgamate, and then readily conclude, or else try to have others conclude, that they cure typhoid fevers, thereby gaining a fictitious notoriety. That class of healers stands between the ten commandments and the code of ethics, so when I hear a doctor say that he cures typhoid fevers, then I mourn for human depravity.

Within the past half century kainomiasmata, as it was then called, was regarded as a contagium located and originating in the cerebro-spinal periphery, the effect taken for the cause, which gave rise to much empirical treatment. When fact takes the place of theory treatment is revolutionized. Theory means talk! Fact means action! Nature is said to be animate and inanimate, and that nothing is annihilated, that chemical changes make up about all that is in nature. The line between we are not prepared to define. So we are unprepared to say whether malaria is the outcome of one or both, whether it be microbic or gaseous in its incipiency. The animalcular theory as propagated by Manson, McCallum, Ross, Laveran and others, who have conducted experiments to prove their theory, has evolved much valuable information. And we trust that chemistry may be invoked to look into the atmospheric and gaseous aspects, that we may look for the initiatory source of miasmatic disorders.

Another separate and distinct idiopathic fever called typhus, or typhoid, is but one disease, and is the outcome of another kind of miasmatic microbe generated from animal effluvia, emanating from animal excreta, and when effectual is capable of infection by contact, and is nowise controlled by heat or cold, prevails in winter or summer, and, unlike the other variety, has no known antidote, but is now anxiously awaiting scientific investigation and inventive genius to reveal, as in the vegetable microbe, a remedy that will destroy typhoid bacillus. To our great allies, chemistry and pharmacy, we look for the antidote. We imagine that we are nearing the time when a remedy will be found, as in the other, to cut short this dread monster that has always been a scourge to humanity, and he who shall make the discovery will have justly a name equal to Jenner.

Up to the present time no known remedy avails either to cure or abort this disease, and all pretenders to the contrary are doomed to failure. No treatment can be relied on, and the experience of ages is to treat typhoid expectantly, and then not to expect too much, because in many cases in which the patient apparently in the beginning had an ugly prognosis, yet he comes out in the end much better than the one who began with apparently a mild case. Your orator, after over a half century's practice and always on the lookout for cause, has almost always found it in either, or all, to-wit: hog-pens, privies, wash-places, or about the dumping-grounds from the culinary departments, and have often seen the spread of this fever arrested as strict sanitary regulations were had, both in and out of the premises.

Typhoid and typhus are synonyms of one and the same disease, whose microbes, according to the discoverer, Laveran, are distinct from that of malaria, and have the same nidus in the blood corpuscles, but are widely different in development and duration, more insidious in movement, time of development and longevity. This fever is often confounded by casual observers with the autumnal estivo-malaria resulting in the unscientific and misapplied name of typho-malarial fever, in which it is supposed coexisted the two diseases, typhoid and malaria, but all evidence at present shows these cases to be simply a pernicious type of malarial fever uncomplicated with typhoid admixture.

The clinical appearance may so nearly simulate typhoid that a correct diagnosis may require an examination of the blood. However, the following points of observation may aid in diagnosis: Herpes is common in autumnal malaria, unusual in typhoid; delirium may appear early in malaria, rare in the early stages of typhoid; abdominal symptoms as well as bronchial are usually early in typhoid, unusual in malaria; anemia is early in malaria, late in typhoid; temperature is more vacillating and covers a wider range in malaria.

It does not legitimately come within the purview of this paper to allude to treatment or therapy, but I can but allude to the diversity of opinion of eminent men in regard to theory and treatment of typhoid fever; both are but poor substitutes for facts, and it is amusing to behold with what tenacity men hold to particular remedial agents that they concoct to suit their special theory, and as typhoid is not amenable to known remedies a wide field is opened for empiricism and empirical remedies, a large proportion of which serve to either kill time or the patient.

---

It is stated that a number of acting assistant surgeons are to be appointed on the army medical corps for service in China and the Philippines, and that the limit of age required of applicants is forty years. Our impression is that heretofore the age limit has been lower than this. If that is the case, we must express our hearty approval of the change.—*New York Medical Journal.*

